# Effects of repetitive transcranial magnetic stimulation on neuropathic pain in patients with spinal cord injury: a systematic review and meta-analysis of randomized controlled trials

**DOI:** 10.3389/fneur.2026.1725648

**Published:** 2026-04-20

**Authors:** Qinghua Luo, Chuansheng Hong, Chunyu Duan, Jiang Ma, Junjie Xiong, Xin Yang, Qimei Jiang, Jingming Hou, Rubing Yan

**Affiliations:** Department of Rehabilitation, Southwest Hospital, Third Military Medical University (Army Medical University), Chongqing, China

**Keywords:** meta-analysis, neuropathic pain, randomized controlled trials, repetitive transcranial magnetic stimulation, spinal cord injury

## Abstract

**Objective:**

The incidence of neuropathic pain following spinal cord injury (SCI-NP) is increasing, and current pharmacological treatments are often limited by severe side effects and diminishing efficacy. This study aims to systematically evaluate the effects of repetitive transcranial magnetic stimulation (rTMS) on SCI-NP and its frequently comorbid emotional disorders.

**Methods:**

We conducted a comprehensive search across five databases (PubMed, Embase, Cochrane Library, Web of Science, and Pedro) up to April 13, 2025. Randomized controlled trials (RCTs) investigating the impact of rTMS on SCI-NP against sham stimulation were included. Pain was the primary outcome, while changes in anxiety and depressive symptoms were secondary outcomes. Data were pooled using standardized mean differences (SMDs) and 95% confidence intervals (CIs).

**Results:**

A total of 131 studies were retrieved from the five databases, and 7 RCTs involving 159 SCI patients were ultimately included. The results indicated that, compared to the control group (sham stimulation), rTMS effectively improved SCI-NP (SMD = −1.41; 95% CI = −2.44 to −0.59; *p* = 0.0007, *I*^2^ = 78%). However, rTMS did not have a significant impact on anxiety (SMD = −0.67; 95% CI = −1.82 to 0.48; *p* = 0.25, *I*^2^ = 66%) or depressive symptoms (SMD = −1.04; 95% CI = −2.26 to 0.19; *p* = 0.1, *I*^2^ = 74%) compared to the control group.

**Conclusion:**

rTMS demonstrates promising potential in alleviating the severity of SCI-NP. However, current evidence does not support a significant therapeutic effect on comorbid emotional states.

**Systematic review registration:**

https://www.crd.york.ac.uk/PROSPERO/view/CRD420251074707, identifier PROSPERO (CRD420251074707).

## 1 Introduction

Neuropathic pain (NP) is a chronic and debilitating complication affecting over 50% of patients with spinal cord injury (SCI) (1, 2). As the clinical incidence of SCI-NP rises, treatment options remain strictly limited. Standard pharmacological interventions—primarily antiepileptics, antidepressants, and opioids—often fail to provide complete relief and are frequently accompanied by significant adverse effects, including excessive sedation, cognitive dysfunction, and addiction with prolonged use (3, 4). Furthermore, SCI-NP is frequently comorbid with mood disorders such as anxiety and depression (5). These psychological factors interact with chronic pain to create a vicious cycle that exacerbates pain perception and profoundly reduces the patient’s quality of life (6, 7). Evaluating pain interventions without considering these emotional comorbidities provides an incomplete clinical picture, underscoring the urgent need for multidimensional therapeutic strategies.

Repetitive transcranial magnetic stimulation (rTMS) has emerged as a promising non-invasive neuromodulation technique (8). Unlike systemic medications, which often provide only temporary relief without addressing the underlying pathology, rTMS delivers targeted magnetic pulses to specific cortical regions (e.g., the motor cortex) to directly modulate the neural pathways involved in pain processing (9, 10). Its primary analgesic mechanisms in SCI-NP are thought to involve the induction of neuroplasticity, the restoration of the disrupted balance between excitatory (glutamate) and inhibitory (gamma-aminobutyric acid, GABA) neurotransmitters, and the modulation of the endogenous opioid system (11, 12). Consequently, rTMS allows for a more precise therapeutic approach with minimal adverse effects, lacking the risks of addiction or tolerance associated with long-term opioid use.

While previous RCTs have investigated rTMS for SCI-NP, the study protocols vary widely, leading to highly discrepant results regarding its efficacy. Given the distinct limitations of current pharmacotherapy, a timely systematic review is necessary. This study aims to objectively evaluate the efficacy of rTMS in alleviating SCI-NP and its impact on comorbid emotional states, synthesizing early-stage evidence to guide future large-scale trials.

## 2 Methods

### 2.1 Protocol and registration

This study was conducted in strict accordance with the PRISMA guidelines (13). Prior to the commencement of the study, we completed the registration on the PROSPERO platform with the registration number CRD420251074707. The data for this systematic review and meta-analysis were obtained from formally published RCTs. This study did not involve the recruitment of patients or the collection of any original patient data, and involves no ethical issues. Therefore, ethical committee approval or review was not required.

### 2.2 Search strategy

The literature search was conducted independently by two researchers. The databases searched included PubMed, Embase, Cochrane Library, Web of Science, and Pedro, with the search covering the time span from the inception of these databases up to April 13, 2025. No language or regional restrictions were applied during the search. The search terms used included “Spinal Cord Injuries,” “repetitive transcranial magnetic stimulation,” “neuropathic pain,” and related keywords. Additionally, manual searches of the databases were performed to ensure that no relevant literature on the study topic was overlooked. After the two researchers completed their individual searches, a third researcher cross-checked the search results and strategies to ensure consistency and accuracy. Detailed records of the search and strategies can be found in Supplementary material 1.

### 2.3 Study selection

All eligible studies were first imported into EndNote (version X9, Clarivate Analytics) for preliminary screening. The screening process was conducted independently by two researchers, who rigorously evaluated each study’s quality and relevance based on predefined inclusion and exclusion criteria. During the preliminary screening, duplicate studies were excluded. Subsequently, the titles and abstracts of all studies were carefully reviewed, and those unrelated to the research topic or with study designs that did not meet the requirements were excluded. Finally, after a thorough reading of the full texts, the studies that met the criteria for inclusion were selected.

After the independent screening by the two researchers, a third researcher reviewed the screening results. In case of disagreements, the researchers discussed the issues in meetings to reach a consensus, ensuring the accuracy and consistency of the included studies.

The inclusion criteria for this study were as follows: (1) only studies involving patients with neuropathic pain after SCI were included. (2) The intervention was rTMS only. (3) The control group received a placebo stimulation. (4) The study must include at least one primary outcome assessment, with the primary outcome being changes in neuropathic pain, and secondary outcomes involving emotional-related outcome measures. (5) The study design must be a RCT.

Exclusion criteria were: (1) Studies with incomplete data or missing key datasets. (2) Studies presenting data exclusively in graphical form without extractable numerical values. (3) Studies in which the intervention group utilized additional adjunctive therapies alongside rTMS, confounding the independent assessment of rTMS efficacy. (4) Studies utilizing a randomized crossover design. rTMS frequently induces long-lasting neuroplastic carry-over effects that are difficult to eliminate during standard washout periods, potentially contaminating the control phase.

### 2.4 Data extraction and management

The data extracted from the included studies consisted of the following information: authors, publication year, country, participant age, interventions used, specific rTMS parameters (frequency, site, pulses, sessions), and outcome measurement standards. Data extraction was conducted independently by two researchers using a predefined template. Once completed, a third researcher verified the data. Any discrepancies were resolved through comprehensive discussion among all three researchers, who referenced the original literature to reach a final consensus. For secondary emotional outcomes, only validated psychometric tools (Hospital Anxiety and Depression Scale [HADS], Beck Depression Inventory [BDI], Hamilton Depression Rating Scale [HAM-D]) were included. Outcome directionality was standardized across all scales prior to pooling, ensuring that higher scores consistently indicated greater symptom severity.

### 2.5 Quality assessment

The risk of bias in the studies was assessed using Review Manager 5.4. This assessment was conducted independently by two researchers, who, after thoroughly reviewing the literature, objectively analyzed and evaluated the potential bias in each study. Upon completion of the assessment, a third researcher reviewed the results to ensure accuracy and consistency in the evaluations.

The focus of this risk of bias assessment was on the following seven areas: selection bias (including random sequence generation and allocation concealment), performance bias (involving blinding of participants and researchers), detection bias (referring to blinding during the outcome assessment), attrition bias (due to missing data), and reporting bias (selective reporting issues). Each area was categorized into one of three risk levels based on the potential for bias: (1) low risk, (2) high risk, or (3) unclear. Through this detailed evaluation process, we ensured a comprehensive and objective judgment of the study quality.

### 2.6 Data analysis

To assess the intervention effects, we calculated the difference between baseline and post-intervention values. The meta-analysis was conducted using Review Manager 5.4. Because the included trials utilized varying measurement tools to evaluate identical clinical outcomes (e.g., Visual Analog Scale [VAS] versus Numeric Rating Scale [NRS] for pain intensity), the standardized mean difference (SMD) and its 95% confidence interval (CI) were calculated.

When assessing heterogeneity between the groups, Cochran’s Q statistic and the *I*^2^ test were used (14). If no significant heterogeneity was found (i.e., the *p* value from the Q test was greater than 0.05 or *I*^2^ was less than 50%), a fixed-effect model was applied for the analysis. Conversely, when significant heterogeneity was present (i.e., the p value from the Q test was less than 0.05 or *I*^2^ was greater than 50%), a random-effects model was used. If the *I*^2^ value exceeded 50%, further sensitivity or subgroup analyses were conducted to explore potential factors contributing to the heterogeneity (14). Statistical significance was determined by a *p* value of less than 0.05.

## 3 Results

### 3.1 Search results

**Figure 1:**
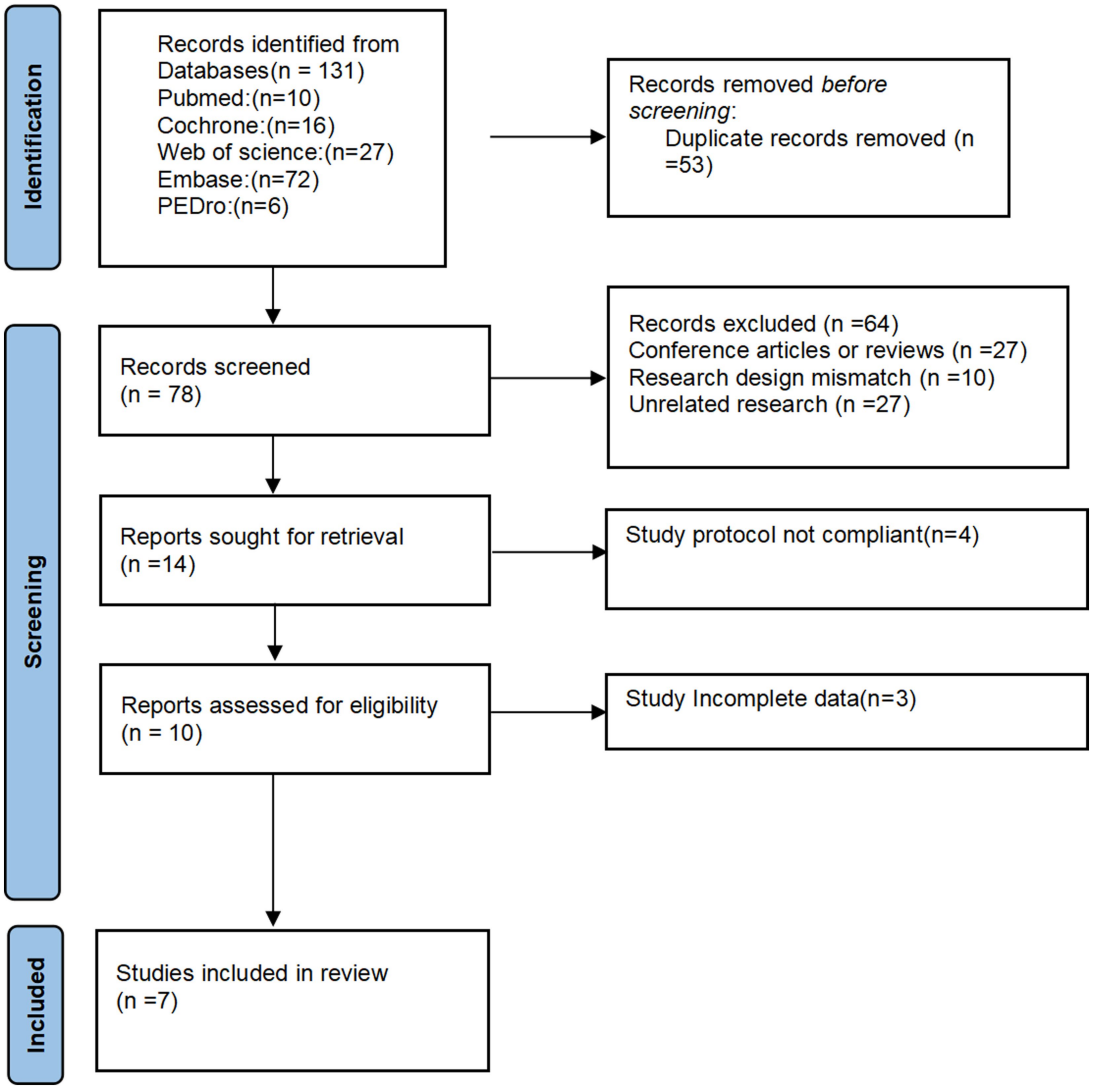
Flow diagram of included studies.

The literature search and screening process is shown in Figure 1. A comprehensive search was conducted across five commonly used medical databases, yielding a total of 131 relevant articles (including 10 from PubMed, 16 from Cochrane, 27 from Web of Science, 72 from Embase, and 6 from PEDro). All retrieved articles were imported into EndNote X9 for management. In the preliminary screening, 53 duplicate articles were excluded, leaving 78 for further evaluation. Subsequently, 27 conference abstracts or review articles, 10 articles with incompatible research designs, and 27 articles unrelated to the research topic were excluded, resulting in 14 articles for full-text review. After a detailed examination of these articles, 4 studies with research protocols not meeting the inclusion criteria and 3 studies with incomplete data were further excluded. Ultimately, 7 studies that met all the inclusion criteria were selected for the final analysis (15–21).

### 3.2 Study characteristics

Table 1 provides a detailed description of the basic characteristics of the included studies. A total of 7 RCTs published between 2007 and 2025 were included, involving 159 SCI patients. All 7 RCTs were published in English. Three studies were conducted in China (16, 19, 21), while the remaining four were conducted in Israel (15), South Korea (17), and Italy (18), and Turkey (20). The intervention group in all 7 studies received rTMS stimulation with different parameters, while the control group received sham stimulation. All 7 studies assessed pain levels before and after treatment, 2 studies evaluated participants’ anxiety levels (16, 18), and 3 studies assessed participants’ depression levels (15, 16, 18). Although different assessment scales were used for each outcome measure, the data corresponding to the same outcome measures were analyzed together.

**Table 1:** Basic characteristics of included citations.

Author, year	Country	E/C(N)	Age (year) (M ± SD)	Experimental group	Control group	Stimulus intensity	Times/weeks of treatment	Outcome
Defrin, 2007 (15)	Israel	5/6	E:56.5 ± 5.89C:54.8 ± 7.66	rTMS	sham	500 trains at 5 Hz for 10s; total of 500 pulses at intensity of 115% of motor threshold.	5/w/2w	PPI, BDI
Jin, 2024 (16)	China	18/15	E:40.7 ± 14.5C:46.5 ± 8.0	rTMS	sham	10 Hz, stimulation duration of 4 s, and interval of 20 s, resulting in 2,000 pulses per treatment session.	5/w/2w	BPI, SAS, SDS
Kim, 2025 (17)	Korea	11/11	E:60.70 ± 9.41C:58.27 ± 20.05	rTMS	sham	A total of 2,000 pulses of 10 Hz rTMS at an intensity of 80% rMT. Each train was composed of 8-s stimulations (80 stimuli), and a total of 25 trains with 10-s intertrain intervals were provided.	5/w/1w	NRS
Nardone, 2017 (18)	Italy	6/6	E:44.33 ± 10.71C:43.50 ± 12.36	rTMS	sham	Each session consisted of 25 series of 5-s pulses of 10 Hz with an interval of 25 s between each train of rTMS, totaling 1,250 pulses per session. The intensity of stimulation was 120% of the resting motor threshold.	5/w/2w	VAS, HAM-A, HAM-D
Sun, 2019 (19)	China	11/6	E:47.00 ± 21.48C:41.00 ± 20.74	rTMS	sham	10 Hz, a total of 1,200 pulses at an intensity of 80% resting motor threshold	6/w/6w	NRS
Yılmaz,2014 (20)	Turkey	9/7	E:40.0 ± 5.1C:36.94 ± 8.0	rTMS	sham	30 trains of 10-Hz stimuli for a duration of 5 seconds at an inter-train interval of 25 s, a total of 1,500 pulses, was applied	5/w/2w	VAS
Zhao, 2020 (21)	China	24/24	41.6 ± 9	rTMS	sham	Active rTMS stimulation was delivered in trains of 15 pulses at 10 Hz (with an intertrain interval of three seconds), for a total of 1,500 stimulations.	6/w/3w	NRS

E = Experimental group; C = Control group; rTMS = repetitive transcranial magnetic stimulation; PPI, present pain intensity; BDI, Beck Depression Inventory; BPI, Brief Pain Inventory; SAS: Self-Anxious Scale; SDS, Self-Depression Scale; NRS, Neuropathic Pain Scale; VAS, visual analog scale; HAM-A, Hamilton Anxiety Rating Scale; HAM-D, Hamilton Rating Scale for Depression.

### 3.3 Quality assessment

**Figure 2:**
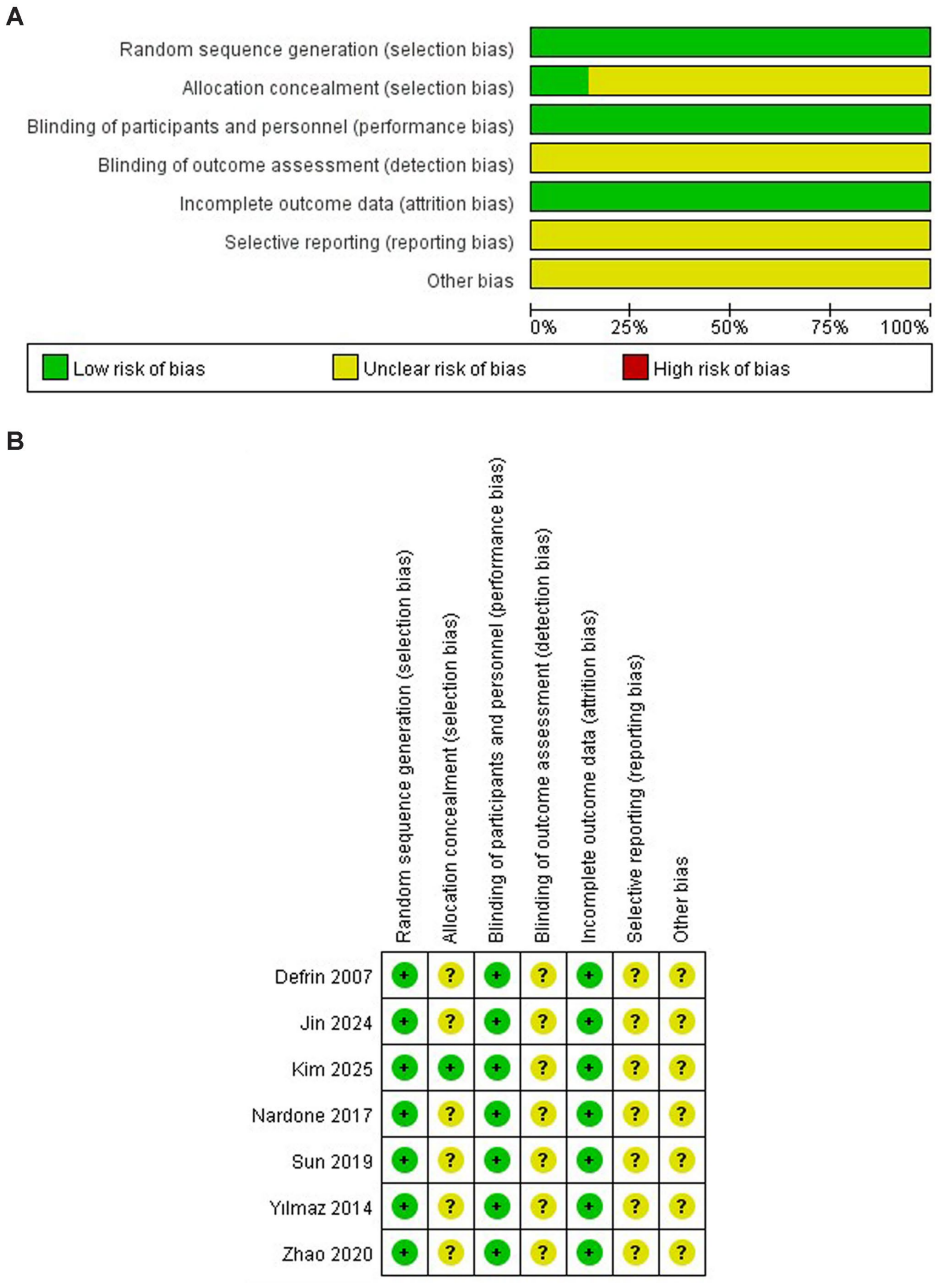
**(A)** Risk of bias graph. **(B)** Risk of bias summary.

The risk of bias for the included studies is shown in Figures 2A,B. All included studies (100%) employed randomization. One study (14.3%) provided a detailed description of the allocation concealment method (17), while the remaining six failed to report their concealment strategy, resulting in an ‘unclear risk’ judgment for this domain. Although all studies implemented blinding for participants and personnel (100%), none explicitly reported whether blinding was applied to outcome assessors, leading to an ‘unclear risk’ of detection bias across all trials. No study was judged to have a high risk of attrition or selective reporting bias, as all provided complete datasets. Given the limited number of included studies (*n* = 7), an assessment of publication bias via funnel plot was not conducted.

#### 3.3.1 rTMS on pain

**Figure 3:**
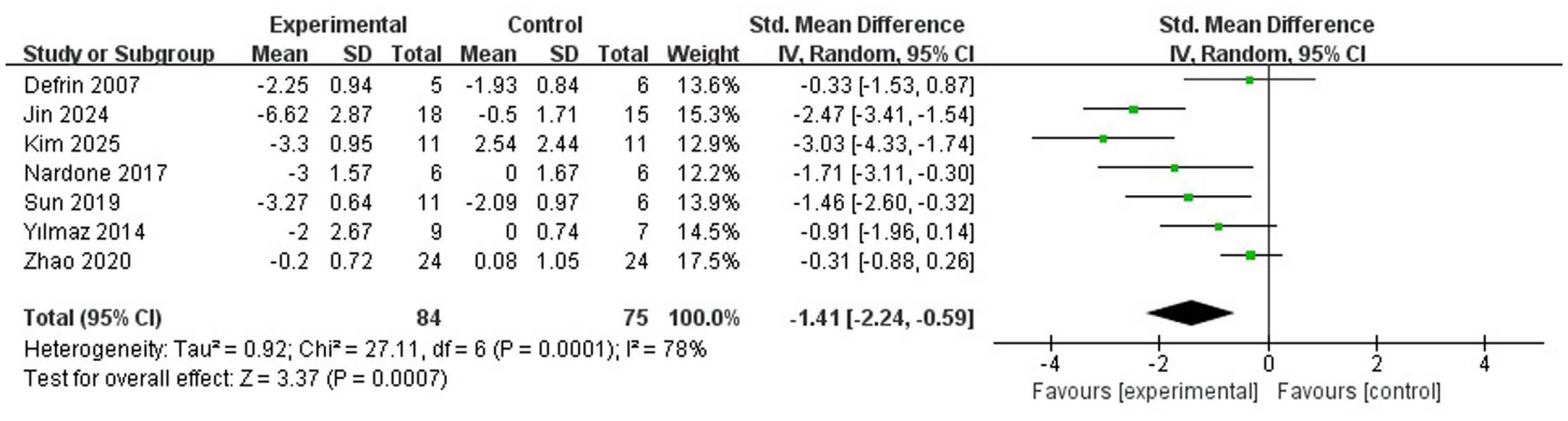
rTMS on pain.

All studies assessed the changes in NP before and after treatment in SCI patients, with a total of 159 participants, as shown in Figure 3. The analysis revealed that compared to the sham stimulation in the control group, rTMS significantly improved SCI-NP (SMD = −1.41; 95% CI = −2.44 to −0.59; *p* = 0.0007, *I*^2^ = 78%). Due to high heterogeneity, a sensitivity analysis was performed, which showed that after excluding two studies (due to multi-target stimulation and mixed acute/chronic pain cohorts) (16, 17, 22), rTMS still significantly improved SCI-NP, with a substantial reduction in heterogeneity (SMD = −0.97; 95% CI = −1.34 to −0.24; *p* = 0.005, *I*^2^ = 34%).

#### 3.3.2 rTMS on anxiety

**Figure 4:**
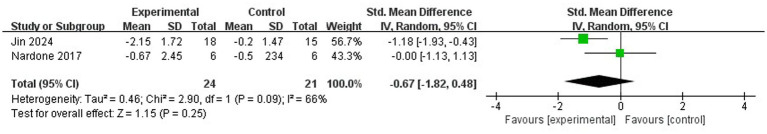
rTMS on anxiety.

Two studies (16, 18) evaluated the changes in anxiety levels before and after treatment in SCI patients, as shown in Figure 4. The results indicated that, compared to the control group, rTMS did not produce a significant difference in alleviating anxiety in SCI patients (SMD = −0.67; 95% CI = −1.82 to 0.48; *p* = 0.25, *I*^2^ = 66%).

#### 3.3.3 rTMS on depression

**Figure 5:**
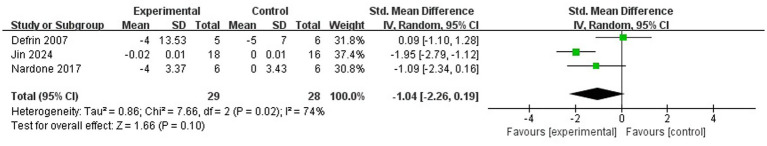
rTMS on depression.

Three studies (15, 16, 18) assessed the changes in depression levels before and after treatment in SCI patients, as shown in Figure 5. The results indicated that, compared to the control group, rTMS did not produce a significant difference in alleviating depression in SCI patients (SMD = −1.04; 95% CI = −2.26 to 0.19; *p* = 0.1, *I*^2^ = 74%). However, after excluding one study (which contributed to substantial clinical heterogeneity), it was found that rTMS significantly improved depression in SCI patients compared to the control group, with reduced heterogeneity (SMD = −1.65; 95% CI = −2.64 to −0.84; *p* < 0.0001, *I*^2^ = 22%).

## 4 Discussion

This systematic review and meta-analysis included seven RCTs of rTMS treatment for SCI-NP, aiming to assess the effects of rTMS on SCI-NP and the mood of SCI patients. No modifications were made to the study protocol after registration. The results of this study indicate that rTMS can effectively reduce the severity of SCI-NP but has no significant effect on the mood of SCI patients. This conclusion is consistent with previous relevant studies (9). However, numerous studies have demonstrated that rTMS may have beneficial effects on patients’ mood, although these studies did not focus on SCI patients, and their potential effects warrant further exploration (23, 24). For example, Li et al. found that rTMS significantly improved the mood state of patients with traumatic brain injury (25). This discrepancy may be due to differences in the type of disease, sample size, or rTMS treatment parameters.

### 4.1 The effect of rTMS on NP

The quantitative synthesis demonstrates that rTMS can effectively alleviate SCI-NP; however, these findings are accompanied by substantial statistical heterogeneity (*I*^2^ = 78%). To explore the sources of this heterogeneity, we conducted a sensitivity analysis. After excluding one study utilizing multi-target rTMS stimulation (16) and another that did not differentiate between acute and chronic pain cohorts (17), rTMS remained effective in alleviating SCI-NP severity, with a notable reduction in statistical heterogeneity.

Furthermore, the residual heterogeneity is highly likely driven by considerable methodological variations across the included trials. Specifically, rTMS protocols differed substantially in stimulation parameters, including frequency (e.g., high-frequency 10 Hz or 20 Hz versus low-frequency protocols), total pulses per session (ranging from 500 to 2000), and overall treatment duration (26, 27). The limited number of available studies precluded formal meta-regression to isolate the specific impact of these parameters, highlighting the optimization of stimulation protocols as a critical area for future research.

In the treatment of SCI-NP, rTMS exerts its effects through multiple pathways and mechanisms, improving patients’ pain perception. The primary mechanisms may include rTMS regulating cortical function in SCI patients to restore neurotransmitter balance. Additionally, some studies have mentioned that rTMS may also promote neuroplasticity and reduce neuroinflammatory responses (28). Following SCI, the first region to undergo pathological reorganization is the cerebral cortex, which includes areas involved in pain perception. When pain signals are transmitted to SCI patients, the cerebral cortex often becomes overly excited, leading to persistent pain that may worsen over time (29). The role of rTMS is to regulate neural activity in these pain perception regions through magnetic stimulation. First, it can promote the inhibitory activity of normal cortical neurons to suppress abnormal neural activity. Second, it can reduce cortical excitability to restore balance (30). In simple terms, rTMS may act on the sensory, motor, and prefrontal cortex regions of SCI patients to inhibit abnormal neural transmission signals caused by SCI and alleviate excessive and prolonged pain (31). The key mechanism here is that rTMS modulates the neural networks of the cerebral cortex, enabling more accurate processing of pain signals and reducing patients’ pain experience.

From another perspective, following SCI, the neurotransmitter system in the spinal cord becomes imbalanced, meaning that the equilibrium between glutamate and gamma-aminobutyric acid (GABA) is disrupted (32). When this balance is disrupted, glutamate becomes overly activated, increasing the excitability of spinal cord dorsal horn neurons in SCI patients and amplifying the transmission of pain signals. Conversely, as an inhibitory neurotransmitter, GABA loses its function when its activity weakens, leading to a reduction or inactivation of the spinal cord’s self-inhibitory mechanism, thereby exacerbating pain in SCI patients (33). rTMS may help SCI patients restore spinal cord excitability balance by regulating the balance between these two neurotransmitters. Previous studies have noted that rTMS can enhance GABA’s inhibitory effects and reduce glutamate activation, thereby alleviating SCI-NP through two complementary mechanisms (34).

The changes in SCI patients are not limited to these. The neural connections between the spinal cord and the cerebral cortex often undergo plastic changes, and rTMS can promote the recovery of neural connections by repeatedly stimulating the cerebral cortex with magnetic fields (35). Notably, rTMS not only improves NP but may also enhance neural regeneration capacity and promote functional recovery after SCI to some extent, though the extent of improvement remains to be explored (36, 37).

Another effect of rTMS is its ability to regulate immune responses, thereby reducing the severity of SCI-NP. This is because the neuroinflammatory response triggered after SCI is a key factor in NP (38). After SCI, local to systemic inflammatory levels significantly increase in patients, leading to elevated levels of pro-inflammatory cytokines such as IL-1β and IL-6, which cause neuronal damage and manifest as persistent severe pain (39). rTMS achieves this through immune modulation, reducing the production of these factors (40). Furthermore, it increases the production and expression of anti-inflammatory cytokines such as IL-10, thereby controlling the inflammatory response (41). By regulating the immune response, rTMS can effectively reduce pain perception and promote neural functional recovery.

### 4.2 The effect of rTMS on emotion

Our findings indicate that rTMS does not demonstrate a statistically significant effect on mitigating anxiety or depression in patients with SCI. Although a post-hoc sensitivity analysis (excluding one trial) yielded a statistically significant improvement in depression scores, this result must be interpreted with strict caution. It serves as hypothesis-generating data rather than definitive evidence of efficacy. The primary objective of the included RCTs was pain reduction; therefore, they were inherently underpowered and not methodologically designed to target emotional disorders.

From the perspective of the target points of rTMS stimulation during treatment, rTMS applies magnetic stimulation to the brain to improve some neural activities in the patient’s cerebral cortex (42). In treating SCI-NP, the target areas are typically selected in regions that can effectively alleviate pain, such as the sensory cortex and motor cortex, thereby prioritizing the relief of pain in SCI patients. However, the brain regions involved in emotional regulation overlap minimally with these pain-related brain regions. These emotional regulation regions include the prefrontal cortex, limbic system, and hypothalamic–pituitary–adrenal axis (HPA axis), among others (43). If rTMS therapy is to be used to reduce SCI-NP while also improving emotional issues in SCI patients, stimulation of the aforementioned brain regions may also be necessary. Differences in therapeutic focus may reasonably lead to variations in efficacy. Targeting only specific regions of the sensory and motor cortices, while rTMS may improve pain, its impact on emotional disorders is limited (44).

Chronic pain, impaired or lost motor function, social isolation from family and society, and loss of confidence in the future can all lead to emotional disorders in SCI patients (45). Prolonged exposure to negative emotions such as anxiety and depression can negatively impact the cooperation and treatment outcomes of SCI patients (46). While rTMS can provide some emotional improvement by alleviating pain, this effect is primarily limited to pain relief itself and does not directly address the patient’s psychological state. Psychological factors, reduced quality of life, and lack of social support can exacerbate emotional issues, and these problems cannot be resolved solely through rTMS.

In theory, rTMS should provide some emotional stability and improvement in pain relief, but the data results did not show significant differences. In the analysis of rTMS effects on depression, after excluding a study with older patients, rTMS significantly improved depressive symptoms in SCI patients with low heterogeneity. Therefore, could there be differences in the efficacy of rTMS across different age groups? Additionally, the final efficacy of rTMS may vary due to individual patient differences, and the location and severity of SCI may also influence treatment outcomes (47). For SCI patients with more severe emotional disorders, the targeted brain regions differ, so rTMS may not fully achieve significant emotional improvement (48).

Emotional issues in SCI-NP still need to be taken seriously. Future research may need to explore treatment models that combine rTMS with other therapies, or synergistic treatment of pain and emotion-related brain regions. For example, rTMS can be combined with cognitive behavioral therapy (CBT) to effectively improve emotional disorders in SCI patients. CBT can help patients better identify and adjust negative thinking patterns, thereby alleviating anxiety and depression symptoms (49). A comprehensive treatment plan combining these methods may provide patients with a more holistic solution, alleviating pain while improving emotional well-being.

### 4.3 Certainty of evidence

Following the Grading of Recommendations Assessment, Development and Evaluation (GRADE) framework, the overall certainty of the evidence synthesized in this review is evaluated as low to moderate. This assessment primarily reflects the inherent landscape of the current literature in this specialized field. Certainty was downgraded due to imprecision—stemming from the relatively small sample sizes characteristic of early-stage trials—and inconsistency, driven by the diverse, unstandardized rTMS protocols across studies (*I*^2^ = 78%). Additionally, incomplete reporting on allocation concealment and outcome assessor blinding in several trials introduces an unclear risk of bias. Despite these limitations, this meta-analysis provides the most comprehensive and highest level of currently available evidence regarding rTMS for SCI-NP. It successfully delineates the promising analgesic potential of rTMS while clearly identifying the specific methodological gaps that future large-scale, standardized trials must address.

## 5 Limitations

This study presents several limitations that reflect the current landscape of this research area. First, the pool of eligible RCTs and their respective sample sizes are relatively small. While meta-analyzing these studies effectively increases the overall statistical power compared to individual trials, the limited total sample size suggests that the calculated effect sizes should be interpreted with appropriate caution. Second, the available data constrained our ability to perform robust subgroup analyses, such as stratifying efficacy by specific rTMS frequencies or cortical targets (e.g., M1 versus multi-target stimulation). Importantly, the included studies encompassed patients with both acute and chronic neuropathic pain. While this provides a comprehensive overview of rTMS in SCI-NP, the underlying pathophysiology differs between these phases, and pooling them may introduce clinical heterogeneity. Finally, as most studies focused on immediate or short-term outcomes, the long-term sustainability of rTMS efficacy remains to be fully elucidated. Despite these limitations, this meta-analysis provides a vital synthesis of the current evidence, offering a valuable foundation to guide the design of future large-scale clinical trials.

## 6 Conclusion

This systematic review and meta-analysis indicates that rTMS demonstrates promising potential as a non-invasive intervention for alleviating neuropathic pain in patients with SCI. However, current data do not support a significant parallel improvement in comorbid emotional states such as anxiety and depression. While the present findings highlight the therapeutic value of rTMS, the current certainty of evidence—influenced by the nascent nature of the primary studies and methodological heterogeneity—suggests that its broad clinical application should be approached with thoughtful consideration rather than immediate routine adoption.

## Data Availability

The original contributions presented in the study are included in the article/Supplementary material, further inquiries can be directed to the corresponding authors.
